# A practical assessment of nano-phosphate on soybean (*Glycine max*) growth and microbiome establishment

**DOI:** 10.1038/s41598-020-66005-w

**Published:** 2020-06-04

**Authors:** Michelle M. McKnight, Zhi Qu, Julia K. Copeland, David S. Guttman, Virginia K. Walker

**Affiliations:** 1 Department of Biology, Queen’s University, Kingston, ON Canada; 2 School of Environmental Studies, Queen’s University, Kingston, ON Canada; 3 Centre for the Analysis of Genome Evolution and Function, University of Toronto, Toronto, ON Canada; 4 Department of Cell and Systems Biology, University of Toronto, Toronto, ON Canada

**Keywords:** Soil microbiology, Plant sciences, Microbial ecology, Nanoparticles

## Abstract

The efficacy of needle-shaped nano-hydroxyapatite (nHA; Ca_10_(PO_4_)_6_(OH)_2_) as a phosphate (Pi) fertilizer was evaluated as well as its impact on soil and soybean (*Glycine max*) bacterial and fungal communities. Microbial communities were evaluated in soy fertilized with nHA using ITS (internal transcribed spacer) and 16S rRNA high-throughput gene sequencing. Separate greenhouse growth experiments using agriculturally relevant nHA concentrations and application methods were used to assess plant growth and yield compared with no Pi (−P), soluble Pi (+P), and bulk HA controls. Overall, nHA treatments did not show significantly increased growth, biomass, total plant phosphorus concentrations, or yield compared with no Pi controls. Soil and rhizosphere community structures in controls and nHA treatment groups were similar, with minor shifts in the nHA-containing pots comparable to bulk HA controls at equal concentrations. The implementation of nHA in an agriculturally realistic manner and the resulting poor soy growth advises that contrary to some reports under specialized conditions, this nano-fertilizer may not be a viable alternative to traditional Pi fertilizers. If nano-phosphate fertilizers are to achieve their conjectured agricultural potential, alternative nHAs, with differing morphologies, physicochemical properties, and interactions with the soil matrix could be investigated using the evaluative procedures described.

## Introduction

With global population anticipated to surpass 9 billion in the next few decades^1^, the demand for phosphate (Pi) fertilizers is also expected to rise in order to maintain sufficient crop production. Elemental phosphorus (P) is obtained by plants in the form of plant-accessible water-soluble Pi salts, which are normally applied to fields as triple super Pi (TSP), and mono and diammonium Pi (MAP, DAP). However, only about 20% of the Pi applied to fields is actually used by the crops during a growing season^2^. Some of the applied Pi forms complexes with soil aluminum, calcium, and iron oxides, resulting in plant-inaccessible forms^3^. However, much of the soluble Pi is lost to agricultural run-off into local water bodies, where it contributes to eutrophication and may cause algal blooms, with their devastating effects on the aquatic ecosystems^4^. Little Pi actually reaches target crops and mineral rock Pi is a limited, non-renewable, and increasingly costly resource^5^. In the future, plant Pi acquisition and use efficiency might be improved^6,7^, but in the short term alternative fertilizing technologies are worth examining.

Nano-hydroxyapatite (nHA; Ca_10_(PO_4_)_6_(OH)_2_) has been proposed as an alternative Pi fertilizer^8^. Nanomaterials have a high surface area to volume ratio suggesting a faster release of soluble ions, compared to bulk materials. Increased soil mobility of nHA could increase root Pi uptake^8^. Thus, nHA should be more efficacious as a fertilizer than bulk HA and indeed, nano-rod HA (hydroxyapatite) was reported to increase the germination rate and growth of sand-grown chickpeas compared with no nHA controls^9^. Spherical carboxymethyl cellulose (CMC) coated nHA promoted the growth and yield of soybeans over that of equimolar amounts of soluble Pi fertilizer, suggesting again that overall, nHA with a higher dissolution rate in nano-form could be a more effective Pi fertilizer, and potentially a more environmentally friendly innovation^8,10^. To date, however, only one study has implemented nHA fertilizer in natural soils, where it was a better Pi source for wheat (*Triticum aestivum*) than bulk HA, but was not more effective than traditional TSP fertilizer^11^. Therefore, although these few results suggested that nHA could be promising, its efficacy has not yet been established when implemented in an agriculturally realistic manner. As well as this concern, no experiments have examined the impact of nHA on plant-associated microbiomes. These microbiomes can play important roles in the maintenance of plant health by inhibiting pathogen colonization and serving as the primary mobilizers of soil nutrients including Pi nutrient acquisition, with some species solubilizing mineralized Pi^12–16^. Some nanoparticles (NPs), including silver and other metal nanoparticles, have been reported to be toxic to soil communities and plants^17,18^, meaning it is possible that the addition of nHA could affect soil and plant microbiome structure, thus it is important to evaluate the impact of novel nano-phosphate fertilizers on both plant and soil microbial communities.

Here, the impact of nHA as a Pi fertilizer on plant growth and microbiome establishment was investigated in *Glycine max* (soybean), which is known for its economically important symbiotic relationship with nitrogen-fixing bacteria *Bradyrhizobium japonicum*. Two independent experiments were conducted: the first to evaluate the effects of nHA on natural soil and plant microbial communities using programmed growth chambers to control for abiotic variables and subsequent high-throughput sequencing methods, and the second to evaluate the efficacy of nHA as a Pi fertilizer under greenhouse conditions. In both experiments, nHA was added directly into the soil at agriculturally relevant concentrations at the time of planting. Overall, our hypothesis was that nHA would not disrupt the soybean microbiomes and would be a promising alternative to traditional Pi fertilizers when implemented in an agriculturally realistic manner.

## Results

### Nanoparticle characterization

**Figure 1:**
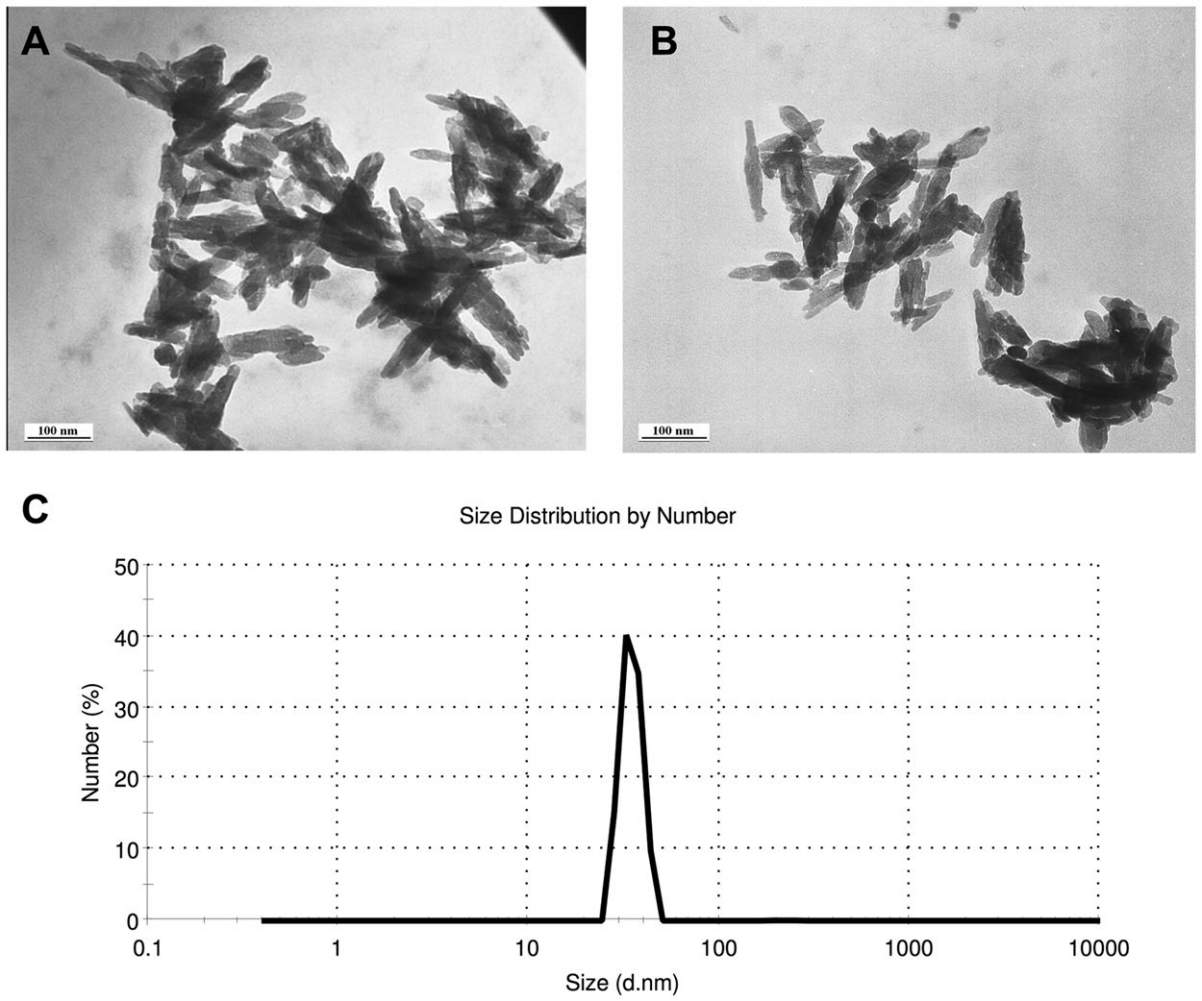
TEM image of nHA sonicated in Milli-Q water (**A**) and in Hoagland’s Solution (**B**), and DLS measurement of nHA suspended in Milli-Q water (**C**). For dynamic light scattering, the size (d.nm) represents the particle distribution in nm.

TEM imaging of nHA showed needle-like particle morphology, with an average width of 23.1 nm (SD + 6.7) and average particle length of 91.7 nm (SD + 29.8) in accordance with the manufacturer specifications (Fig. 1A,B). Similar particle characterization was seen when nHA was suspended in Milli-Q water or Hoagland’s Solution, with evidence of some agglomeration even after sonication (Fig. 1A,B). Dynamic light scattering (DLS) analysis showed a mean peak at 34.9 nm (Fig. 1C), again similar to the size range seen in the TEM images.

### nHA microbiome experiments

#### Effect of microbiome experimental conditions and treatments on soybean growth

**Figure 2:**
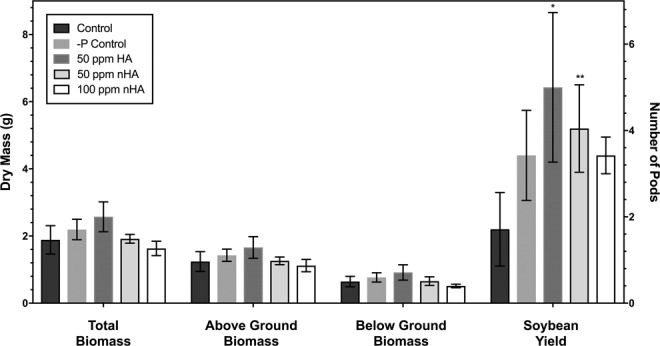
Additional soy grown for 8 weeks during microbiome experiment were analyzed for total biomass, pod number, above ground biomass, and below ground biomass. Average biomass and pod number is shown (n = 5), with error bars representing standard deviation. No significant difference was found in above, below, or total biomass between treatments when tested using a one-way ANOVA (*P* > 0.05). Stars above the bars indicated a significantly higher pod number in the HA 50 ppm (*P < 0.05) and nHA 50 ppm (**P < 0.01) compared with control treatment.

Microbiome experiments were performed in controlled growth chambers, consisting of five treatments: control (A), −P control (B), HA bulk control (C), 50 ppm nHA (D), and 100 ppm nHA (E) (Table 1). Soy was grown in a 5% farm soil mixture to provide natural microflora to promote microbiome establishment. Along with plants grown for microbiome sample collection (n = 20 per treatment), additional plant replicates were grown under each treatment (n = 5 per treatment) to measure the effect of treatment on plant growth and yield (Table 1). There were no treatment effects on the number of active root nodules harvested after 8 weeks of growth (n = 5, *P* = 0.99) with the average number of nodules present on roots for treatments A-E at 13.2 (SD + 6.4), 14.0 (SD + 5.4), 13.6 (SD + 3.8), 13.0 (SD + 4.2), and 13.2 (SD + 4.8) respectively. In treatments B-E, 100% of nodules had a reddish-pink interior, consistent with active nitrogen fixation. Host plants also showed no visual signs of foliar nitrogen deficiency, nor were there any differences in above ground biomass (*P* = 0.81), below ground biomass (*P* = 0.08), or total biomass (*P* = 0.09) between treatments (Fig. 2). Treatments C and D with added HA and nHA at 50 ppm, showed a significantly higher number of pods when compared with control treatment A (*P* < 0.05) (Fig. 2), although this was not apparent for the high nHA (100 ppm) treatment E (*P* > 0.05). To demonstrate the ability of the soy variety to show a healthy phenotype under non-limited Pi conditions, an additional supplementary experiment including a soluble Pi treatment was performed under the same conditions used for the microbiome experiment (see Supplementary Information). This showed that under the microbiome experimental conditions, soluble Pi addition did promote a significant increase in plant growth and yield, compared to −P control (B) and 100 ppm nHA treatment (E) (Table S1).

**Table 1:** List of all 5 treatments used for the nHA microbiome experiment with replicate number and added solutions. Additional growth replicates were included during microbiome experiment to assess growth of plants under experimental conditions.

	Treatment	Final Concentration	Phosphorus Concentration**	Microbiome Replicates	Growth Replicates	Added Solution
A	Control	—	—	20	5 + 3 extra	Water
B	−P Control*	—	—	20	5 + 3 extra	KNO_3_ & Hoagland’s***
C	HA Bulk Control (50 mg/kg)	50 ppm (4.3 mg HA)	9.25 ppm P	20	5 + 3 extra	KNO_3_ & Hoagland’s***
D	nHA (50 mg/kg)	50 ppm (4.3 mg nHA)	9.25 ppm P	20	5 + 3 extra	KNO_3_ & Hoagland’s***
E	nHA (100 mg/kg)	100 ppm (8.6 mg nHA)	18.5 ppm P	20	5 + 3 extra	KNO_3_ & Hoagland’s***

*P control contains no added Pi to the soil either at time of planting or in fertilizing solution.

**Concentration of P based on equivalent moles of P added in either 50 or 100 ppm of HA or nHA in 86 g of soil.

***Hoagland’s solution contains no N or P.

#### Bacterial community analysis

For both 16S and ITS amplicon sequencing, 10 of the 20 replicate plants from each treatment were randomly selected for analysis of soil, rhizosphere, and phyllosphere/endosphere microbiomes. Sequencing of the V4 hypervariable region of the total 150 leaf, soil, and root samples (with an additional three sequencing plate control samples), yielded 8,933,913 reads and 929 unique operational taxonomic units (OTUs) with the mean number of reads per sample at 58,391 (standard deviation, SD = 28,188). Plate positive controls had >99.9% of reads assigned to *Pseudomonas*, whereas the negative controls contained only two reads. The filtered OTU table was parsed by sample type; soil, root, and leaf.

Phyllosphere/endosphere sequencing was performed using peptide nucleic acid (PNA) clamps, which had been previously reported to reduce amplification of host plant plastid and mitochondrial DNA, which have homology to bacterial 16S rRNA gene sequences^19^. After initial quality filtering, there were 929 OTUs (total of 3,515,900 reads) across all 50 leaf samples (Table S2), with 99.9% belonging to plastids (Fig. S1A). After filtering these, 1662 reads remained (Table S2). Although an underlying microbiome structure composed of 14 different phyla was revealed (Fig. S1B), overall low read numbers cautioned against further analysis. The specificity of the PNA clamps must be increased in future studies in order to assess the upper vegetative portion of soybeans for changes in community structure.

**Figure 3:**
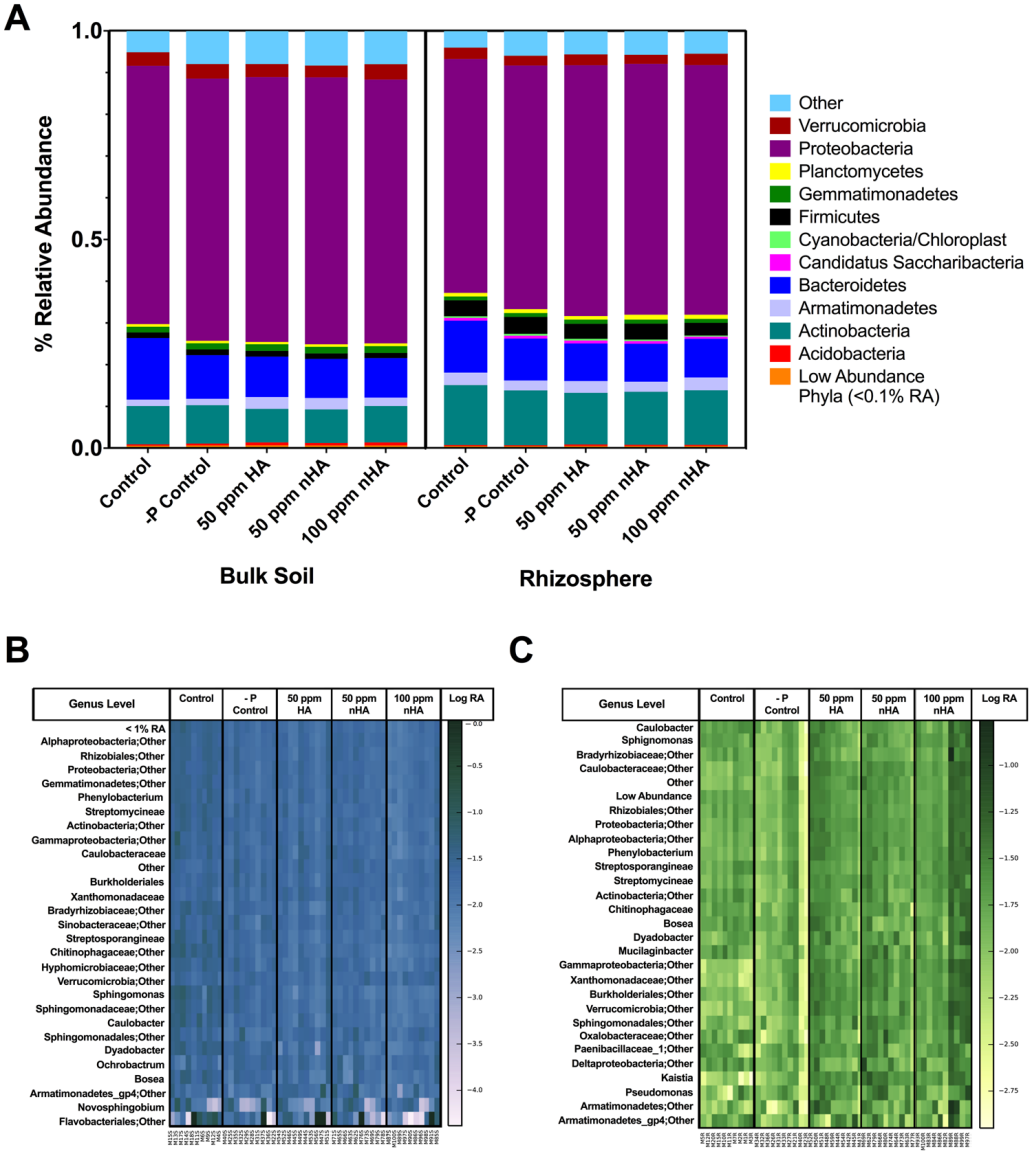
Average relative abundances of phyla in each treatment group for both bulk soil (left) and rhizosphere samples (right) from microbiome experiment (**A**), and heatmaps of highly abundant taxa (>1% RA) at the genus level for both bulk soil (**B**) and rhizosphere (**C**) samples. Overall, the abundance of taxa across all samples is very similar. The rhizosphere samples show a high taxonomic diversity between samples in each treatment, with no visible pattern of increase or decrease in any taxa occurring within treatments. Heatmaps display taxonomic data as log normalized relative abundance at the genus level.

Average relative abundances of various taxa at the phylum level in the soil or rhizosphere communities showed high similarity between the −P control (B), 50 ppm HA (C), 50 ppm nHA (D), and 100 ppm nHA (E) treatments, with some modest difference apparent in the −P control treatment for both soil and rhizosphere samples (Fig. 3). These differences in the relative abundances of certain phyla were confirmed using non-parametric ANOVA analysis, which showed a significant decrease in Bacteroidetes in the −P control, 50 ppm HA, 50 ppm nHA, and 100 ppm nHA treatment samples compared with the untreated controls in both the rhizosphere and soil (Tables S3 and S4). There was a lower relative abundance of the taxa assigned to ‘Other’ phyla in the control treatment, compared to the other four treatments (both soil and rhizosphere) and a significant increase in soil Acidobacteria in treatments that contained HA or nHA (50 and 100 ppm) when compared to the untreated controls. Both Firmicutes and Actinobacteria in the rhizosphere samples showed an increased abundance compared to soil (Fig. 3A). Below the level of phyla, when differences in relative abundance were examined at the genus level, there was a high similarity of abundance across all HA and nHA treatments (Fig. 3B,C). Overall, statistical analysis of the high abundance genera (>1%) showed some significant effects, however notably, there were no striking significant differences in genera present in the 50 ppm HA and 50 ppm nHA treatment groups.

**Figure 4:**
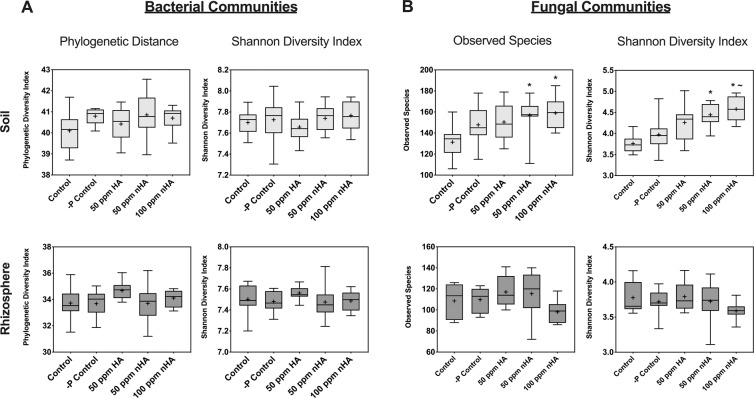
Alpha diversity measurements of bacterial (**A**) and fungal (**B**) communities for microbiome experiment. Bacterial alpha diversity was calculated using phylogenetic distance (PD) and Shannon index for both soil and rhizosphere grouped by treatment. Fungal alpha diversity was calculated using Shannon index and observed species in both soil and rhizosphere samples. Calculations of alpha diversity metrics were performed on OTU data rarefied to the minimum sample count, and statistical tests were done using a non-parametric one-way ANOVA with Dunn’s multiple comparison test to determine significant differences between treatment pairs. Crosses indicate the mean value within each treatment, while the middle line within the box indicates the median value. An asterisk indicates a significant difference compared to control treatment and tilde indicates a significant difference compared to −P control treatment (*P* < 0.05).

Alpha diversity estimation used rarefied OTU data and calculated both phylogenetic diversity (PD), which uses phylogenetic relationships, and the Shannon index as a measure of combined species richness and evenness. Across all treatments in both soil and rhizosphere samples, neither PD nor Shannon index showed differences between treatments (Fig. 4A). This was confirmed using a non-parametric one-way ANOVA (n = 10, *P* > 0.05).

**Figure 5:**
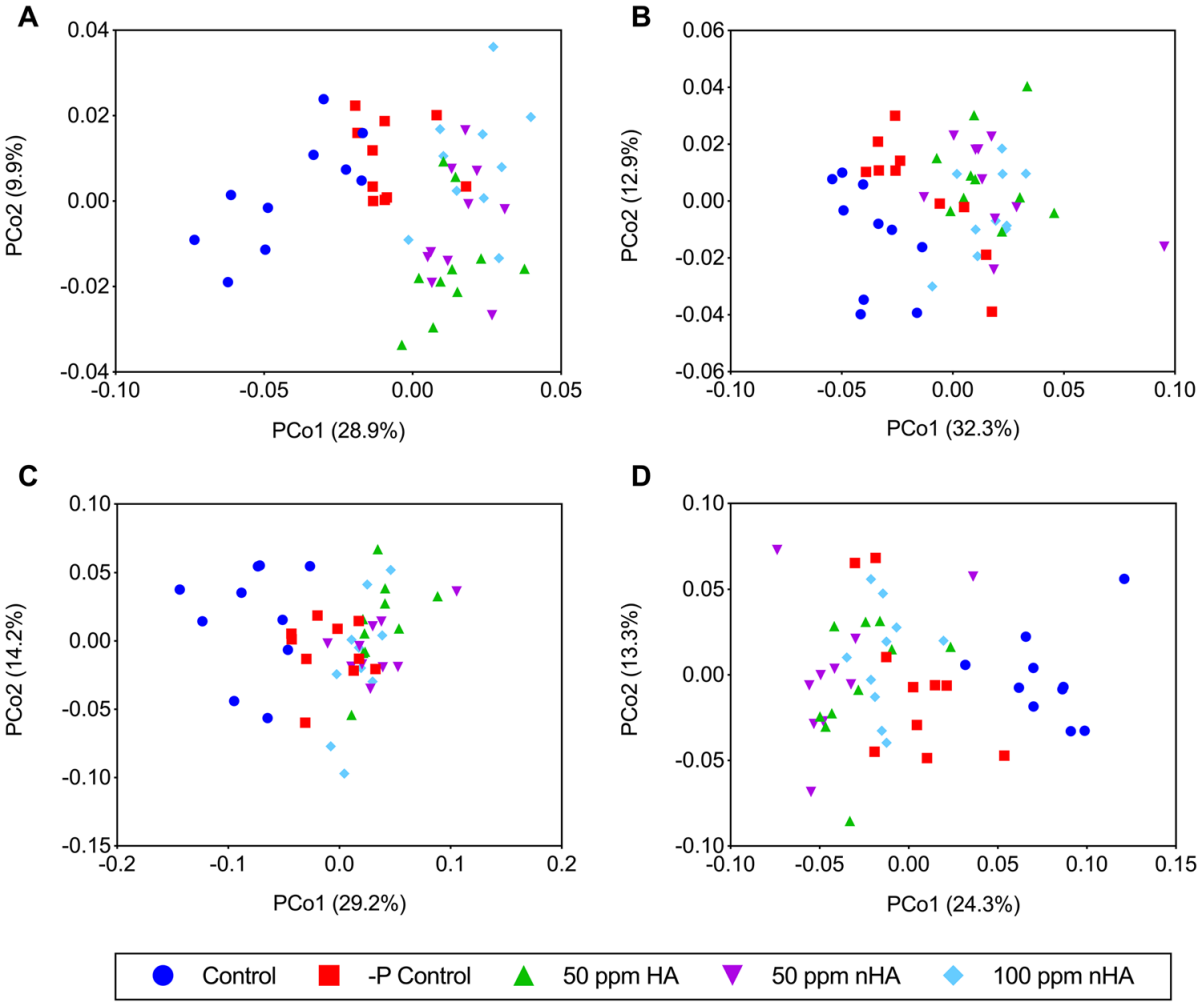
Principle coordinates analysis (PCoA) of beta diversity in bulk soil and rhizosphere using the weighted UniFrac metric. Samples were normalized both via CSS normalization (**A,B**) and rarefied to the minimum sample count (**C,D**) prior to beta diversity calculations. Bulk soil PCoA plots for both methods of normalization (**A,C**) appear similar, with clustering occurring in the control treatment samples and −P control treatment samples. Similar clustering patterns can be observed in both of the rhizosphere PCoA plots (**B,D**) and the soil plots (**A,C**).

Beta diversity was calculated using the weighted UniFrac beta diversity metric, which considers phylogenetic relationships and the proportion of different OTUs present in different samples. Calculations were performed on both rarefied and cumulative sum scaling (CSS) normalized OTU tables. Because rarefaction discards usable data^20^, CSS normalization was also included^21^. PCoA was used to visualize any separation between samples and treatments, and these plots showed a similar pattern between treatments in both the soil and rhizosphere samples and were consistent using both methods of normalization. Markedly, control treatment samples clustered separately from the 50 ppm HA and nHA, and 100 ppm nHA treatments, whereas the −P control samples clustered between the controls and the nHA/HA samples (Fig. 5). To determine if the visible clustering between treatments was indeed significant, both adonis (PERMANOVA) and ANOSIM non-parametric tests were performed (Table 2). Both tests indicated significant separation overall between treatments for both soil and rhizosphere samples, using weighted UniFrac beta diversity distances calculated using OTUs from both methods of normalization (*P* < 0.001). To confirm clustering, non-parametric t-tests were conducted between each pair of treatments, using Bonferroni correction to adjust *P*-values for multiple comparisons. Significant differences between the control treatment and the 50 ppm HA, 50 ppm nHA, and the 100 ppm nHA treatments were confirmed indicating that nHA/HA treatments had an impact on beta diversity in both the soil and rhizosphere samples (Tables S5 and S6). It is worth emphasizing however that there was no difference in beta diversity of the soil microbial communities, whether the hydroxyapatite was present in bulk or nano form. Although there was a significant difference found between the beta diversity distance within the 50 ppm HA treatment, compared to the distance between the 50 ppm HA treatment and 100 ppm nHA treatment (*P* = 6.12E-06) for CSS normalized data, there was no significant difference when comparing beta diversity distance within the 100 ppm nHA treatment to the distance between samples from the 100 ppm nHA treatment and 50 ppm HA treatment (*P* > 0.05) (Table S5). The differing results between comparisons likely comes from the greater dispersal of samples within the 100 ppm nHA treatment compared to the closer clustering of samples within the 50 ppm HA treatment (Fig. 5A). Additionally, there is no significant difference in beta diversity distance between these two treatments when rarefied data are analyzed (Table S5). Along with the observed overlap in data points between the 50 ppm HA and 100 ppm nHA, the data do not indicate the 100 ppm nHA treatment had a major impact on the soil bacterial community structure. Further experiments with bulk HA controls at the same concentration as 100 ppm nHA would be required to confirm whether any small effect from the addition of 100 ppm nHA compared to 50 ppm HA was the result of exclusively nHA addition and not just the addition of HA, regardless of its form.

**Table 2:** Results of non-parametric statistical tests adonis (PERMANOVA) and ANOSIM testing the effect of treatment on beta diversity distance for both soil and rhizosphere, rarefied and CSS normalized OTU data.

Normalization	Type	Test	Test statistic	*P* value
Rarefaction	Soil	ANOSIM	0.337	0.001
		adonis	0.298	0.001
	Root	ANOSIM	0.280	0.001
		adonis	0.245	0.001
CSS Normalization	Soil	ANOSIM	0.458	0.001
		adonis	0.324	0.001
	Root	ANOSIM	0.344	0.001
		adonis	0.271	0.001

#### Fungal community analysis

**Figure 6:**
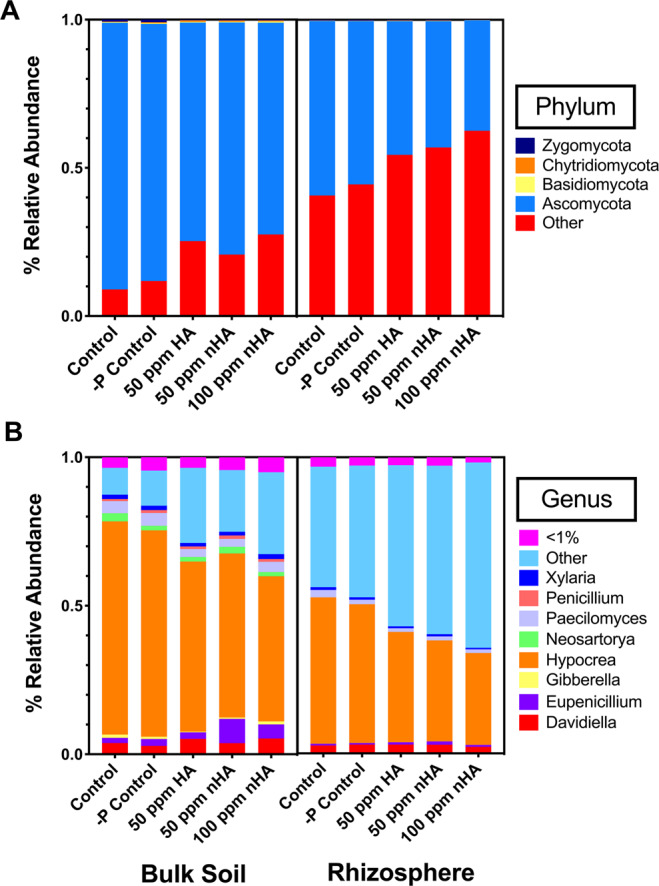
Average relative abundances of fungal phyla in each treatment group for both bulk soil (left) and rhizosphere samples (right) (**A**), and average relative abundances of highly abundant fungal genera (>1% RA) in both bulk soil (left) and rhizosphere samples (right) (**B**).

Sequencing of the ITS-1 region resulted in 812,810 and 970,173 reads for fungal bulk soil and rhizosphere communities, respectively, with an average of 16,256 (SD 6,180) and 19,404 (SD 9842) reads per sample respectively. On the level of phyla, there was a significant decrease in soil Ascomycota for 50 ppm HA, 50 ppm nHA, and 100 ppm nHA treatments, accompanied by an increase in OTUs assigned to ‘Other’ phyla when compared with the control treatment (*P* < 0.05) (Fig. 6A, Table S7). The same pattern was observed in the rhizosphere, but only when comparing the 100 ppm nHA treatment to controls (*P* < 0.05) (Table S8). At the genus level in both bulk soil and rhizosphere, the majority of significant changes were detected in rarer genera (<1%) and showed no pattern across treatments (i.e. changes consistent with addition of HA/nHA or fertilizer) (Tables S9 and S10). As observed at the phylum level, the same increase in relative abundance of ‘Other’ genera was also noted (Tables S9 and S10) (*P* < 0.05). The most notable change at the genus level for both the bulk soil and rhizosphere was in *Hypocrea*. This filamentous fungus, a member of the Ascomycota and well known for their production of secondary metabolites, was found in greater relative abundance in the soil compared to the rhizosphere community, irrespective of treatment. However, there were significant decreases in *Hypocrea* in the 50 and 100 ppm nHA treatments compared with the control treatment (*P* < 0.05). This change was notable as *Hypocrea* had the greatest relative abundance within the soil community across all treatments, ranging from ~50–70%, while in the rhizosphere, *Hypocrea* is present with the second highest relative abundance, ranging from ~30–50% across all treatments.

Using non parametric one-way ANOVA (Kruskal-Wallis), alpha diversity assessments of the rhizosphere fungi showed no significant difference in the richness and evenness between all 5 experimental treatments, or the number of observed OTUs, estimated using the Shannon index (χ^2^ = 7.7, n = 10, *P* = 0.10) and observed species (χ^2^ = 9.5, n = 10, *P* = 0.05) metrics respectively (Fig. 4B). However, within the soil fungal communities, there were significant differences in these parameters (Shannon index, χ^2^ = 25.5, n = 10, *P* < 0.0001 and observed species, χ^2^ = 14.8, n = 10, *P* = 0.005). Using Dunn’s post-hoc test, a significant increase in observed species was found in the 50 ppm and 100 ppm nHA treatments when compared with the controls (*P* < 0.05) (Fig. 4B). A similar pattern was noted for richness and evenness, with a significant increase in the 50 ppm nHA treatment (*P* < 0.05) and 100 ppm nHA treatment (*P* < 0.01) when compared with the control treatment, and as well a significant increase in the 100 ppm nHA treatment was observed compared to the −P control treatment (*P* < 0.05).

When beta diversity was assessed, samples did not clearly cluster into discrete treatment groups in either the bulk soil or rhizosphere communities for both methods of normalization used (Fig. S2). However, samples from both control groups (control and −P control) appeared to overlap, while samples from the 50 ppm HA, 50 ppm nHA, and 100 ppm nHA also overlapped, but less obviously than the controls. Nevertheless, statistical analysis on beta diversity distance matrices using both non-parametric tests ANOSIM and adonis revealed significant dissimilarity between groups (*P* < 0.01), but test statistics at below 0.25, suggested this was a low dissimilarity between treatments (Table S11). When beta diversity distances between samples from a single treatment were compared to their distance from samples of another treatment, results using different methods of normalization (CSS and rarefaction) were not always consistent. For example, although rarefied data did show significantly smaller beta diversity distances within the 100 ppm nHA treatment compared to its distances from the four other treatments (*P* < 0.05; Fig. S2), CSS normalized data in the rhizosphere showed significantly smaller beta diversity distances amongst the −P control treatment compared with all four other treatments.

### nHA growth and production experiments

#### Plant growth

**Figure 7:**
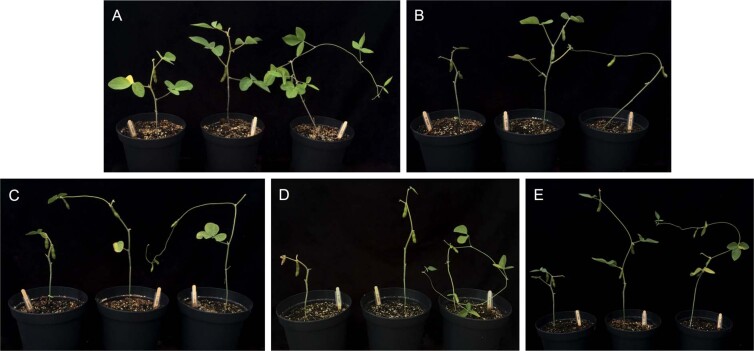
Range of phenotypes (lowest, mean, and highest growth shown in left, middle, and right pots, respectively) for each treatment, with respect to height, pod, and trifoliate number during greenhouse growth and production experiment (GP2). Photographs were taken 10 weeks after planting for control treatment (**A**), −P control (**B**), HA (**C**), nHA corresponding to 15 ppm P (**D**) and nHA corresponding to 56.5 ppm P (**E**).

**Figure 8:**
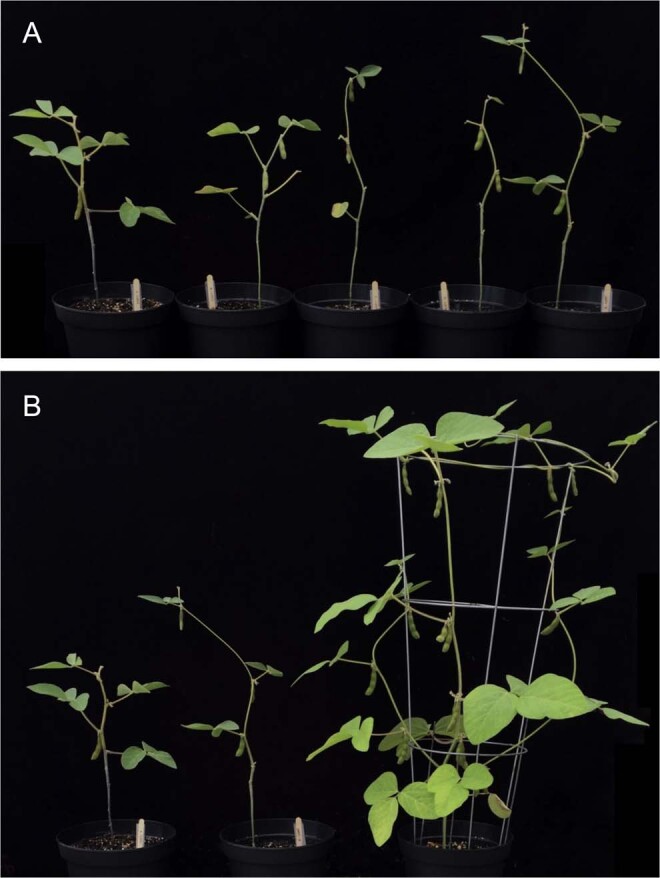
Mean phenotypes from each treatment group during greenhouse growth and production experiment (GP2) with treatments A-E for control, −P control, HA, nHA (15 ppm P), and nHA (56.5 ppm P) respectively (left to right) (**A**) and comparison of average plants from control treatment A (left), nHA at 56.5 ppm P in treatment E (middle), and +P control plants treated with standard Pi fertilizer (right) (**B**). Plants shown representing the average phenotype were selected based on height, pod number, remaining leaves, and total number of trifoliate leaves.

**Figure 9:**
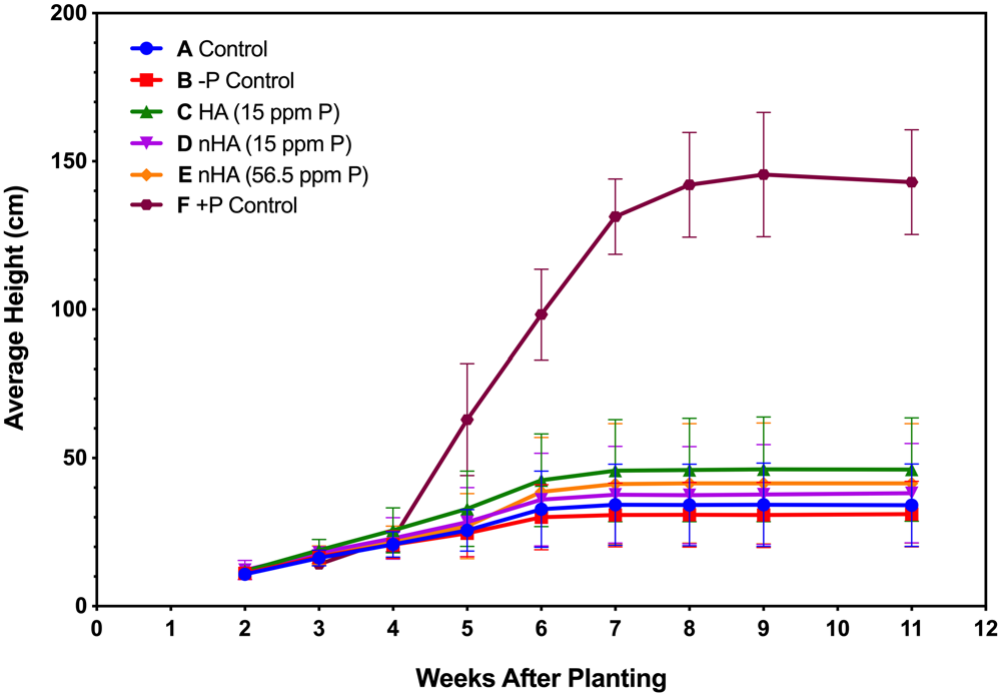
Height measurements starting at week 2 after planting during greenhouse growth and production experiment (GP2), taken weekly for all 5 treatments (treatments A-E representing controls, −P treatment, HA and 15 ppm and 56.5 ppm concentrations of nHA, n = 20) as well as standard Pi fertilizer (treatment F, n = 5). Error bars represent standard deviations.

Separate treatments (5) were used to assess the effectiveness of nHA as a Pi fertilizer (Table 3). Control soybeans were grown in the absence of any fertilizer (treatment A). Treatment B plants were given fertilizer without any added source of P or Pi, and treatment C was given HA at the same concentration as the treatment D group. Two treatment groups (D and E) contained nHA as a source of Pi at two different agriculturally relevant concentrations (Table 3). Greenhouse experiments were repeated twice, with a first experimental replicate (GP1), followed by a second experimental replicate (GP2). An additional treatment group (F), containing plant usable Pi as the source of P, was included in the second experimental replicate (GP2) and used as a positive P control to demonstrate the growth and seed potential of the soy cultivar under full nutrient conditions. Treatment F was also repeated on its own under the experimental conditions at a later date, demonstrating the same results observed during GP2 experiment. GP1 and GP2 experiments yielded similar results in that there was no increase in plant growth observed in the nHA amendments for GP1 and 2 when compared with the −P control treatment groups. Because the results were consistent, only the second experimental replicate (GP2) serves as representative data here, as it also includes the additional positive P control (F) (Table 3). Although there was some phenotypic variation within treatments, overall there was little difference in plant appearance across all 5 treatments (A-E) 10 weeks after planting, including height, trifoliate leaves, and pods produced (Figs. 7 and 8A). However, plants fertilized with soluble Pi (treatment F) showed striking increases in the above ground biomass and pod number compared to other treatment groups (Fig. 8B). Heights (recorded weekly) over the course of 12 weeks were also similar for treatment groups A-E where growth slowed at around 7 weeks. In contrast, soluble Pi amendment plants (F) continued to grow for 1–2 additional weeks (Fig. 9). As a consequence, the final mean height (38.2 cm) for plants from treatment groups A-E were not significantly different (*P* > 0.05), whereas the average height (143 cm) of positive P control plants was significantly greater (*P* < 0.0001). It should also be noted that the unexpected presence of thrips resulted in some foliar damage to the newly emerged seedlings (Fig. S3), but the minor damage was irrespective of the treatment group as determined by non-parametric one-way ANOVA (*P* > 0.05). The subsequent application of biocontrols successfully reduced thrip numbers to such a low level that little to no foliar damage visible on the newly emerged leaves, with surveys showing 2.6% of plants with a live thrip present (~1.5 individuals per plant). The lower number of standard Pi-fertilized plants grown in the second trial (GP2, treatment F) during the second trial was due to the unavailability of additional biocontrols, but additional plants were grown later when thrips had been eradicated from the greenhouse.

**Table 3:** Summary of five treatments used for the two replicate greenhouse growth and production experiments GP1(first replicate experiment) and GP2 (second replicate experiment), including additional positive control (F) in GP2 experiment.

	Treatment	Soil Additions	Replicates	Added Solution	Experimental replicate including treatment
A	Control	—	20	Water	GP1, GP2
B	−P Control*	—	20	Plant-Prod 14-0-14	GP1, GP2
C	HA (15 ppm P)	32.4 mg of HA	20	Plant-Prod 14-0-14	GP1, GP2
D	nHA (15 ppm P)	32.4 mg of nHA	20	Plant-Prod 14-0-14	GP1, GP2
E	nHA (56.5 ppm P)**	153 mg of nHA	20	Plant-Prod 14-0-14	GP1, GP2
F	Soluble Pi (+P control)	—	5	Plant-Prod 20-20-20	GP2

*P control contains no added Pi to the soil either at time of planting or in fertilizing solution.

** Final concentration of P in soil was based on recommended application rate of 20 kg/ha P_2_O_5_.

**Figure 10:**
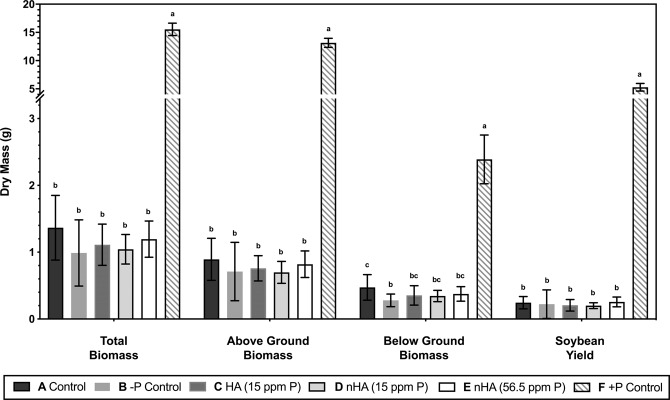
Average total biomass, soybean yield, below ground biomass, and above ground biomass for different treatment groups from greenhouse growth and production experiment (GP2) including controls, −P controls, HA at 15 ppm P, nHA at 15 ppm P, nHA at 56.5 ppm P, and soluble Pi additions (treatment groups A-F, respectively) Bars represent mean values with error bars showing standard deviation (n = 20 treatments A-E, n = 5 treatment F). Treatments with a common letter superscript above are not significantly different, as determined via TukeyHSD post-hoc test (α = 0.05).

At 12 weeks’ post planting there were no significant differences between treatment groups A-E with average total and above ground biomass across treatments at 1.1 g (SD + 0.4 g) and 0.8 g (SD + 0.3 g), respectively. However, there was a significant increase in both total and above ground biomass, 15.5 g (SD + 1.1 g) and 13.2 g (SD + 0.8 g) respectively, for treatment F compared with all of the other five treatment groups (*P* < 0.0001) (Fig. 10). The same pattern was observed for the average seed yield, with a significant increase in seed production when plants were treated with standard Pi fertilizer (with a mean of 5.3 g seeds/plant) compared to the other five treatments, which were similar (Fig. 10) and together averaged 0.23 g of seeds (*P* < 0.0001). This result is also reflected in the number of seeds and pods, with the only significant increase in number seen treatment F, compared to the other treatment groups (Table 4). Below ground biomass results (Fig. 10) showed a similar pattern in that the average below ground biomass for treatment F was 2.39 g, which was significantly different from the mean biomass of 0.36 g for treatments A-E (*P* < 0.0001). However, there were some differences amongst treatment groups A-E. The −P control treatment had a significantly lower below ground biomass compared with the control treatment (*P* = 0.001) in both experimental trials. Overall, nHA treatments did not show a significant increase in biomass or yield when compared with controls, with the only significant increase in growth and yield seen mediated by the addition of soluble Pi as indicated.

**Table 4:** Average number of seeds and pods measured during growth and production experiment GP2 across treatments A-E (n = 20) and treatment F (n = 5), with average concentration of total plant P (µmol/g) in above-ground soy tissue (n = 3). Standard Pi fertilizer treated plants (treatment F) were compared with treatments A-E using TukeyHSD post-hoc analysis following one-way ANOVA that showed significant increases in seed, pod number, and plant P concentration in treatment F compared to all other treatments (*P* < 0.0001). Mean values with common superscript letters are not significantly different based on post-hoc analysis (α = 0.05).

	Treatment	Average Seed Number	Average Pod Number	Plant P Concentration (µmol/g)
A	Control	3.2 + 0.9^b^	1.9 + 0.6^b^	4.9 + 0.9^b^
B	−P Control	3.4 + 1.7^b^	2.5 + 1.1^b^	8.1 + 1.7^b^
C	HA (15 ppm P)	3.0 + 1.0^b^	2.1 + 0.8^b^	8.3 + 2.7^b^
D	nHA (15 ppm P)	2.7 + 0.8^b^	2.3 + 0.6^b^	7.3 + 0.7^b^
E	nHA (56.5 ppm P)	3.4 + 1.1^b^	2.5 + 0.6^b^	6.6 + 0.8^b^
F	Soluble Pi (P_2_O_5_)	41.2 + 9.1^a^	20.0 + 1.6^a^	148.0 + 16.5^a^

Analysis of the total plant P content of above ground plant tissue showed the same pattern as observed in the analysis of overall biomass. Not unexpectedly, plants treated with soluble Pi had a significantly higher average concentration of P in their above ground tissues than the treatment groups A-E (*P* < 0.0001), which averaged 148.0 µmol/g DW (dry weight) and 7.1 µmol/g DW respectively (Table 4). This suggests that nHA was no better at providing the plant with P than the bulk HA and provided no more P to the plant than was available in the control and −P control. Soil samples from treatments A-E, collected at the end of the growth trial all had soluble Pi concentrations <0.1 µmol/g DW.

## Discussion

Nano-fertilizers are currently available commercially, however questions remain about their impact on plant microbiome establishment and their nutrient efficacy when applied in an agriculturally realistic manner. Prior to examining these two questions, the nano-phosphate particles used in these experiments were characterized and were confirmed as nano-sized (~23 nm × ~92 nm) and needle-like in shape. Elongated nHA was chosen because of its high surface area to volume ratio, with the rationale that they would maximize the rate of dissolution of soluble Pi ions from the surface of the particles. There was some agglomeration, even after sonication in both Milli-Q water and Hoagland’s solution. Such NP association was also observed in the nHA used to test the efficacy of nHA as a fertilizer in P sorbing soils^11^.

Overall, the application of nHA had no major impact on soil and rhizosphere bacterial and fungal communities and minor changes observed in the nHA amended communities compared to controls were mostly noted in treatments with bulk HA. Significantly, the formation of active nitrogen-fixing nodules by *B. japonicum* in soy roots was not impacted; after 8 weeks there was no effect on the number of nodules formed on soybean roots suggesting that nHA does not interfere with this symbiotic relationship. Nodules were internally reddish-pink in colour, consistent with active nitrogen fixation. Control plants (treatment A) receiving only water, had nodules but with no evidence of active nitrogen fixation, likely due to the absence of sufficient N prior to the V2 stage, the point at which soy seedlings form active nodules^22^. There was no visual evidence, including leaf colouration that suggested the other plants (−P control, HA or nHA treated) had a N-deficiency that could lead to reduced growth, although these experiments did not allow us to make any conclusions regarding rates of nitrogen fixation, only the establishment of actively N-fixing nodules. In contrast, signs of P-deficiency including thin, spindly stems, small leaves, and early chlorosis of lower leaves^23^, were exhibited by plants in all these treatment groups, and demonstrating that the addition of farm soil to the nutrient poor soil mix did not result sufficient Pi levels for vigorous growth, nor did the addition of HA or nHA to the soil (Figs. 7–9).

### Effect of nano-phosphate (nHA) on soybean microbiomes

As indicated above, the symbiotic relationship with *B. japonicum* was not perturbed by nHA. However, for bacterial microbiome communities, HA and nHA amendment was correlated with a significant decrease in Bacteroidetes in both the rhizosphere and soil (Tables S3 and S4; but see below) and a significant increase in Acidobacteria in treatments that contained HA or nHA, when compared to the untreated controls. In contrast, for the fungal community, most of the significant differences between treatments were found for low abundance genera, except for *Hypocrea* (teleomorph *Trichoderma*)^24^. Members of this genus, *T. ghanese*, are known to have plant growth-promoting abilities and reduce certain diseases in rice^25^. Thus, the significant decrease in relative abundance in this genus after 100 and 50 ppm nHA treatment compared to the control and −P control treatments may be of concern, however, additional controls would be needed to determine if changes in relative abundance are due to nHA, any form of HA, or nutrient solution (without P and N). As for the bacterial communities, there was no overall effect on fungal community structure in both the bulk soil and soybean rhizosphere after the addition of nano-phosphate compared to the bulk HA.

To reiterate, there were differences in fungal community richness and diversity noted between HA, nHA, and control treatment groups in the bulk soil microbiomes, although these changes were not observed in the rhizosphere communities. There were no significant differences in bacterial alpha diversity between treatments, but beta diversity analysis of bacterial communities showed clustering by treatment groups, with control samples significantly clustered apart from all three treatments containing HA (nano or bulk). This indicates that the experimental treatment groups were associated with a small, but significant shift in the overall microbial community in the soil. Because the relative abundance of Bacteroidetes in soil communities receiving Hoagland’s solution (treatments B-E) decreased, this suggested that soluble nutrient addition may be responsible. Previously, decreases in Bacteroidetes have been noted in soil communities with ammonium addition compared to unfertilized plots^26^, and after adding N and P to grassland soils^27^. By comparison, sample clustering by treatment group was less evident in the rhizosphere communities, which could be attributed to selection by host plants^28^. Generally, as indicated, there was an increased relative abundance of Firmicutes and Actinobacteria in the rhizosphere compared to soil communities across all treatments (Fig. 3A). Similarly, Firmicutes abundance increased in black soybean rhizosphere soils relative to bulk field soils, and in winter rye (*Secale cereale L*.) plant growth-promoting Actinobacteria was observed to colonize the rhizosphere and rhizoplane during inoculation experiments^29,30^. These results, which are consistent with field studies, argue that we were successful in inoculating sterilized laboratory soils.

Although our results show that HA/nHA has a minor impact on soil microbial communities, importantly, there was no difference in effect on community structure between the nano or bulk form of HA at the observed concentration of 50 ppm HA or nHA (Fig. 5). This suggests that amendments with these NPs do not result in a dramatic reduction in the relative abundances of phyla beneficial for plant health, as has been seen with the application of metal NPs in a variety of soils^17,18^. Significantly, however, for the assessment of the utility of nano-fertilizers, the lack of change in the microbial communities from the nHA treatment group compared with the bulk HA treatment group suggests that there was no more soluble Pi released by the nHA than by the bulk HA. When concentrations of available P (in the form of Pi) are high in the soil, usually due to fertilizer amendments, shifts in soil microbial community composition and microbial activity can result, especially in P-deficient environments^31^. We concede that no conclusions can be made on the impact of nHA on the physiological activity within the soil and rhizosphere microbiomes. In this regard, it is likely that any available Pi would be used by the soy, with the result that Pi levels may have been too low to impact microbial Pi pools, although additional experiments are required to test this hypothesis; here there was no evidence that experimental plants were getting large amounts of Pi from the nHA, as they exhibited many symptoms of Pi deficiency, as noted below.

### Effect of nano-phosphate (nHA) on soybean growth and production

Both experimental greenhouse trials indicated that nHA was not an effective source of Pi when added directly to the soil at agriculturally comparable concentrations (low = 15 ppm P of nHA and high = 56.5 ppm P of nHA). Neither biomass nor yield increased significantly, compared with control treatments (Fig. 10, Table 4). However, when seedlings were given soluble Pi (treatment F), they were significantly taller, had a higher biomass and yield, appeared more robust, and showed no premature leaf chlorosis^23^, compared to all the other 5 treatment groups, which all showed evidence of Pi deficiency (Figs. 9 and 10). Pi deficiency is supported by assays of total P concentrations in above ground plant tissue in all treatments (A-E), which were <8 µmol/g, in contrast to the average concentration in the positive P treatment of ~145 µmol/g, which indicates the nHA is not effective at supplying adequate Pi to the plant. Low concentrations of soluble Pi in the soil (<0.1 µmol/g) amended with nHA also suggests that the nHA was ineffective in releasing sufficient amount of Pi ions into the surrounding soil to provide the plants with, at minimum, the critical concentration of available P in the soil required for growth. Thus, these results also support our observations that nHA was not an effective source of P to prevent P-deficiencies in the plants.

In contrast to the results reported here, previous studies showed that spherically-shaped nHA was a more efficient source of Pi compared with traditional soluble fertilizers for soybean and lettuce, respectively^8,32^. It is possible that this difference could be attributed to the shape of the nHA, as well as the regular solution applications used, which would not be practical for farmers. Notably, we applied needle-like nHA once in the soil at seeding time, as would be used in agricultural practice. In a 2015 study, nHA with a similar needle morphology was reportedly a more effective P source for wheat, *Triticum aestivum* than bulk HA, however it did not have the efficacy of soluble Pi^11^. In the highly P-sorbing andisol and oxisol soils used for the study, nHA had a higher mobility than HA^11^. Again, these authors applied nHA and HA suspended in solution, and thus we suggest that nHA addition to the soil at the time of planting may explain why our ‘nano-fertilizer’ behaved more like bulk HA, and why there was similar clustering of microbial communities in the nHA and HA treatment groups from the microbiome experiment (Fig. 5). Increased rate of nHA dissolution compared to bulk HA, or possibly uptake of nHA particles by the roots is hypothesized to be the mechanism by which these nanoparticles make Pi more bioavailable to the plants^11^. If the nHA was behaving more similarly to its bulk counterpart, then increased particle size could decrease the dissolution rate and particle mobility in the soil, decreasing the bioavailability of Pi to the plant.

## Conclusion

Overall our hypothesis that nHA would not affect soybean microbiomes and would be a promising alternative to traditional Pi fertilizers could not be accepted. When nHA was implemented in an agriculturally realistic manner, with a moderate rate of application, it did not function effectively as a source of Pi. This is an important result since we recognize that nano-fertilizer is currently available to some farmers. As well, because of the promise of nHA to extend rock Pi resources, more efficiently fertilize crops, and reduce field run off leading to eutrophication^10^, it is a priority to practically test these claims. It is heartening that there was no evidence of negative effects on the soy microbiome or nodulation, however, nHA was not efficacious at concentrations recommended for traditional Pi fertilizers. We hope that the results presented here will inspire new practical application technologies that will be more effective, through further investigation into other nanoparticle morphologies and application methods used, allowing these nano-fertilizers to fulfill their promise.

## Methods

### Nanoparticle characterization

Although nano-fertilizers are advertised for sale to growers from several companies in China and India, full details of their compositions were not available. Thus, characterized hydroxyapatite (Ca_10_ (PO_4_)_6_(OH) _2_) nanoparticles (nHA) were purchased from Sky Springs Nanomaterials, Inc. (Houston, Texas, USA) as <40 nm, with a needle shaped morphology, and 98.5% purity. nHA shape and size was confirmed using transmission electron microscopy (TEM) after suspension in Milli-Q water and sonication for 10 min, with mean nHA length determined using ImageJ^33^. Sonicated Milli-Q water suspensions of nHA were used to verify particle size distribution and average size by DLS analysis using a Malvern Zetasizer Nano ZS.

### nHA microbiome experiment

#### Farm soil mixture

Peat-pearlite SunGro Sunshine® Mix #2 soil with no added fertilizer (soluble Pi < 10 µg/g^34^) was sifted to remove large twigs and autoclaved twice to ensure sterility. It was then inoculated with field soil (5% w/w) obtained from a farm north of Sydenham, ON (44.4°N, 76.6°W) which had not been treated with fertilizer for 25 years. The soils were thoroughly mixed using a cement mixer for 15 min, bagged, and incubated at 22–25 °C until further use.

#### Experimental treatments and cultivation

A total of 140 pots (13 cm) were used, consisting of treatments with 20 pots each sampled for microbiomes (n = 100), 5 replicate pots used for monitoring growth under experimental conditions (n = 25), and three extra replicates per treatment (Table 1). Additional +P control pots fertilized with soluble Pi were monitored separately (Table S1, see Supplementary Information).

Each pot contained 86 g of the soils (95:5 sterile potting soil and farm soil, respectively, and incubated to “seed” the mixture with field-derived microorganisms). HA and nHA additions were made at the concentrations indicated (treatments C-E; Table 1) in a depression (3 cm in diameter and 1 cm deep) made in the soil mixture (Fig. S4A). Three soybean seeds (Wallace Variety, Willow Agriservice, Harrowsmith, ON) were planted 1 cm below the soil surface around the centre of the pot (Fig. S4A). The seeds had been previously coated with HiStick® N/T Soybean co-inoculant (containing *Bradyrhizobium japonicus* and *Bacillus subtilis* MBI600) at the recommended rate (3.2 mg/g of seed). After covering seeds and nHA/HA with soil, pots were watered with reverse-osmosis (RO) water (200 mL, as determined empirically). Pots were placed in a growth chamber set to 23–24 °C during the day, 20 °C at night, with a photoperiod (estimated at 600 mE/m^2^s^1^) of 13 h^35^. Plants were rotated within the chamber every other day to minimize place effects. One week after planting, the seedlings were thinned so that one plant remained per pot. Treatment groups B-E were fertilized with 50 mL of 3.5 m*M* KNO_3_ solution to facilitate the formation of nodules for the first week after planting^22^, and subsequently fertilized every other day with 50 mL of Hoagland’s solution lacking nitrogen and phosphorus (see Supplementary Methods, Table S12)^36^. Treatment A pots were provided with a comparable 50 mL of RO water daily, with all plants in each treatment group watered every other day with RO water as required.

The 5 growth replicates per treatment were cultivated for 8 weeks and fertilized as described. Above ground and below ground plant tissues were then collected, dried (48 h at 70 °C), and weighed. Pods and root nodules were counted. Nodules were dissected to determine reddish-pink colouring, an indication of active nitrogen fixation^37^.

#### Sample collection

After three weeks, all plants had reached the V2 stage (2^nd^ trifoliate stage) and samples from the leaf, root, and soil were collected for microbiome analysis. From each of the 20 plants per treatment the middle leaf of the first trifoliate was removed with ethanol-sterilized forceps and kept at 4 °C for ~2 h in a sterile bag until processed. Four cores were then aseptically removed from different areas of each leaf using a flame-sterilized cork borer (1 cm diameter), placed in a 1.5 mL centrifuge tube, snap frozen with liquid nitrogen, stored at −80 °C, and designated as the endosphere/phyllosphere (above ground on and within plant microbiome).

Soil samples were collected using a scoopula sterilized with 70% ethanol from the center of each pot, and after removing the top 1 mm of soil, enough soil was collected from each pot to fill a 15 mL conical centrifuge tube. Samples were stored at −20 °C until they were lyophilized prior to extraction. Roots were gently and aseptically separated from the remaining soil and stored at 4 °C in sterile bags overnight. A 2.5 cm root section was cut with sterilized scissors 2.5 cm down from the stem-root junction, and subsequently any adhering nodules and lateral roots were removed from the root section samples. The root samples were placed in 1.5 mL centrifuge tubes containing 1.3 mL of Pi-buffered saline (PBS) containing 1 m*M* EDTA and 0.01% Tween-20, and shaken for 20 min at 180 rpm, vortexed for 15 sec and then transferred into a new tube. The remaining root wash solution, containing any microbial cells and DNA washed from root surface, was centrifuged (20 min at 335 × g) and the supernatant removed, with the remaining pellet frozen in liquid nitrogen and stored at −80 °C and designated as the rhizosphere sample.

#### DNA extraction and electrophoresis

Soil and rhizosphere DNA samples were extracted using the NucleoSpin® Soil Genomic DNA Extraction Kit (Macherey-Nagel) following the manufacturer’s protocol but with the addition of a repeated sample lysis step (SL1 lysis buffer and Enhancer SX). For soil samples (150 mg), 150 μL of extra lysis buffer was used. Pelleted rhizosphere material (50–290 mg; mean 125 mg) was initially suspended in SL1 lysis buffer and transferred to bead tubes with the rest of the extraction carried out following the manufacturer’s protocol.

Phyllosphere/endosphere DNA was extracted with the Wizard® Genomic DNA Purification Kit (Promega, WI, USA) following the manufacturer’s protocol for the isolation of plant genomic DNA, with an added lysozyme step^38^. All DNA extractions were quantified using both Nano-Drop and Qubit® Fluorometer (Invitrogen, ThermoFisher). DNA quality was also verified using 1% agarose gel electrophoresis with samples stored at −20 **°**C before further analysis. Prior to sequencing, community structure of the bulk soil was examined using PCR-denaturing gradient gel electrophoresis to verify the presence of a detectable bacterial community (not shown).

#### rRNA and ITS gene amplicon sequencing and analysis

For each of the 5 treatments from the bulk soil, rhizosphere, and phyllosphere/endosphere samples, 10 out of the 20 replicate DNA samples from each treatment were randomly selected for 16S rRNA and ITS gene amplicon sequencing (150 total). DNA samples were sent to the Centre for the Analysis of Genome Evolution & Function (CAGEF) at the University of Toronto for sequencing. The V4 hypervariable region of the 16S rRNA bacterial gene fragments were amplified using a universal forward sequencing primer (515 F) and a uniquely barcoded reverse sequencing primer (806 R) to allow for multiplexing^39^. Amplification reactions for the soil and rhizosphere samples were performed using 12.5 μL of KAPA2G Robust HotStart ReadyMix (KAPA Biosystems), 1.5 μL of 10 μ*M* forward and reverse primers, 8.5 μL of sterile water and 2 μL of DNA. The V4 region was amplified by cycling the reaction at 95 **°**C for 3 min, 18x cycles of 95 **°**C for 15 sec, 50 **°**C for 15 sec and 72 **°**C for 15 sec, followed by a 5 min 72 **°**C extension. For the peptide nucleic acid (PNA) clamp reactions using leaf DNA extract as the template, 2 μL of DNA was added to 12.5 μL of KAPA2G Robust HotStart ReadyMix (KAPA Biosystems), 1.5 μL of 10 μ*M* forward and reverse primers, 6 μL of sterile water, 0.75 μL of 25 μ*M* plastid PNA clamps and 0.75 μL of 25 μ*M* mitochondrial PNA clamps (PNA BIO Inc., Newbury Park, CA). The V4 region was amplified by cycling the reaction at 95 **°**C for 3 min, 20x cycles of 95 **°**C for 15 sec, 78 **°**C for 15 sec, 50 **°**C for 15 sec and 72 **°**C for 15 sec, followed by a 5 min 72 **°**C extension. The fungal ITS1(internal transcribed spacer) region was amplified using ITS1F and ITS1R primer sets^40^ as described for the V4 procedure but by reducing the DNA template to 1 μL and optimizing amplification with 25 cycles of 95 °C for 15 sec, 56 °C for 15 sec and 72 °C for 15 seconds, followed by a 5 min 72 °C extension.

For both bacterial and fungal DNAs, all amplification reactions were done in triplicate as well as negative controls for each barcode, verified on a 1% agarose TBE gel, and then pooled to reduce amplification bias. Pooled triplicates were combined by approximately even concentrations as determined by a Qubit fluorometer. Standard Nextera XT protocols were followed, selecting for 300–500 bp fragments, with the final libraries purified using 0.8X magnetic Ampure XP beads, again quantified using fluorescence (Qubit) and each of the bacterial and fungal libraries were separately pooled and sequenced, according to manufacturer’s instructions (Illumina MiSeq, San Diego, CA). For both library sets, sequencing was performed using V2 (150 bp × 2) chemistry

The UNOISE pipeline, available through USEARCH version 9.2, was used for sequence analysis^41–43^. The last base, which is typically error-prone, was removed from all the sequences. Sequences were assembled and quality trimmed using –fastq_mergepairs and –fastq_filter set at 1.0, with a –fastq_maxee set at 0.5. Sequences less than 233 bp (20 bp shorter than the average) were also removed. For fungal ITS analysis, following the UNOISE pipeline, unique sequences were identified from the merged pairs and for all analysis, merged pairs were de-replicated and sorted to remove singletons. Sequences were denoised and chimeras were removed using the unoise2 command. For 16S rRNA gene analysis, assembled sequences were mapped back to the chimera-free denoised sequences at 97% identity OTUs. Taxonomy assignment was executed using utax and the UNOISE compatible Ribosomal Database Project (RDP) database version 16, available to download through USEARCH, with a minimum confidence cutoff of 0.9^44^. OTU sequences were aligned using PyNast accessed through QIIME^45^. Sequences that did not align were removed from the dataset and a phylogenetic tree of the filtered aligned sequence data was made using FastTree^46^. For ITS analysis, assembled sequences were mapped back to the chimera-free denoised sequences at 97% identity OTUs using the –otutab command. Taxonomy assignment was executed using SINTAX^43^, available through USEARCH, and the SINTAX compatible Ribosomal Database Project (RDP) Warcup ITS v2 database, with the default minimum confidence cut-off of 0.8^44^.

Prior to analysis, both bacterial and fungal OTU tables were filtered to remove any OTUs represented by sequences numbering less than 0.005% of the total^47^. Any unassigned OTUs were also removed prior to analysis. The OTU tables were separated based on sample type to create individual OTU tables for soil, rhizosphere, and leaf samples (endosphere/phyllosphere), as applicable, which were then analyzed separately. The majority of the subsequent OTU table analysis was done using QIIME (1.9.1)^45^, including data normalization, taxonomic relative abundance, rarefaction, alpha diversity calculation, and beta diversity calculations. Alpha diversity was calculated on data rarefied to the minimum sample counts using both Faith’s phylogenetic diversity index (PD) and the Shannon index for 16S rRNA gene analysis, while observed species (OS) and Shannon index were used for fungal ITS data. Prior to beta diversity analysis, data were normalized using both rarefaction to the lowest sample count and also cumulative sum scaling (CSS) normalization^21^. Beta diversity was calculated using the weighted UniFrac metric, which takes into account the taxonomic relationship and abundance of OTUs within samples^48^. Principle coordinates analysis (PCoA) and generation of heatmaps were done using QIIME.

### Statistical analysis

Statistical analysis of plant biomass and pod numbers was done using one-way ANOVA and TukeyHSD post-hoc tests calculated through GraphPad Prism (version 7.0, GraphPad Software, La Jolla, CA, USA). Non-parametric ANOVA (Kruskal-Wallis test) run through R (version 3.3.2) was used to determine significant differences in phylum and genus relative abundance between treatments, with post-hoc testing done using Dunn’s multiple comparisons test implemented through the FSA package in R. Differences in alpha diversity were also tested using non-parametric ANOVA, here implemented through GraphPad Prism. Analysis of the statistical significance of sample groupings for beta diversity metrics was done using the compare_categories.py script in QIIME, which uses the R vegan package to run both ANOSIM and adonis (PERMANOVA) tests using 1000 permutations. Finally, statistical analysis of beta diversity distances within and between treatments was done using the make_distance_boxplots.py script in QIIME, which ran parametric t-tests with Bonferroni *P*-value correction for multiple comparisons. All statistical tests were conducted using a significance level (α) of 0.05.

### Growth and production experiments

#### Experiment set-up

Six treatments (A-F) were set up in greenhouse experiments (Table 3). Dry peat-pearlite (500 g) SunGro Sunshine® Mix #2 (contains no added fertilizer, and not autoclaved as microbiomes were not examined) was placed into 100 pots (20 cm diameter) and then wetted with RO water. Additions were made to the pots as required (treatments C-E) in the centre of each pot. A 50 mL conical centrifuge tube was used to lift a core of soil 7.6 cm deep, below which the HA or nHA was placed before replacing the overlaying soil. Soybean seeds (3) as previously described, were planted 2.5 cm below the surface surrounding the centre of all pots (Fig. S1B) and watered. Two weeks after planting, seedlings were thinned to one per pot, with un-germinated seeds also removed from the pots. Notably, in these experiments, soybean seeds were surface sterilized by successively immersing them for 1 min in 70% ethanol, sterile water, 10% sodium hypochlorite, sterile water and subsequently rinsed 3X with sterile water before potting. Surface sterilization was intended to remove or kill fungi or bacteria, including nitrogen-fixing bacteria, as experimental treatments supplied the nitrogen. For treatment groups C-E (Table 3), HA or nHA was placed 5 cm below the soybean seeds in the centre of the pot in accordance with agricultural recommendation as well as fertilizer concentrations as suggested for soybean (Ontario Ministry of Agriculture, Food and Rural Affairs; OMAFRA). Levels of 20 kg/ha P_2_O_5_ represent the lowest recommended field application rate when the soil test level indicates a P concentration of 13–15 ppm^49^. Thus nHA/HA was added in treatment E to provide equimolar amounts of P in the form of Ca_10_(PO_4_)_6_(OH)_2_ dictated by the recommended application rate of 20 kg/ha P_2_O_5_ based on the surface area of the 20 cm pots used for this experiment. The lowest concentration of P in the soil recommended to grow soy is 15 ppm, which is considered the critical P concentration below which crops experience decreased yield due to P-deficiencies^50^. This concentration of 15 ppm P in the form of HA or nHA was used in treatment C and D respectively, based on the dry mass of soil used in the pots. This concentration was informed by the previous reports indicating that nHA was more effective than soluble P fertilizers (P_2_O_5_) at the same rate of application^8^.

Soy was grown in a greenhouse (20–24 °C with a photoperiod of 18 h). The first replicate experiment (GP1) was conducted for 12 weeks (late April-July), with a second replicate experiment (GP2) similarly conducted (mid-May- late July). Two weeks after planting and approximately one week after germination, 350 mL Plant-Prod® 14-0-14 Balance Fertilizing solution was applied weekly to treatments B-E (Table 3) at a rate of 100 ppm N with 350 mL of RO water given weekly to treatment A. Treatment F was given 350 mL of Plant-Prod® 20-20-20 weekly also at a rate of 100 ppm N. Additional water was applied equally to all treatments as required. Soy growth was monitored weekly for the duration of the experiments. Height was recorded weekly and the number of trifoliate leaves, seed pods, and amount of foliage lost was recorded for each plant.

During GP1 replicate growth, thrips were observed in the greenhouse, resulting in mild foliar damage 10 days after planting. Subsequently, predatory Swirski-Mites (Koppert Biological Systems) biocontrols were applied once every fortnight to each plant and thrip presence was monitored as recommended by a modified version of Purdue University’s protocol^51^. Briefly, each of the four greenhouse benches was demarcated in 5 zones, from which a trifoliate leaf (GP1: uppermost trifoliate; GP2: lowest trifoliate) from 5 randomly selected plants were enumerated for the presence of thrips at any life stage.

#### Sample collection for biomass and yield

After 12 weeks of growth, all GP1 and 2 plants were harvested. The above ground portion of the plant was cut from the roots ~1 cm above the first lateral root and placed in a paper bag for drying. Roots were carefully separated from the soil, washed with water and bagged as previously described. They were then dried for 2 days at 70 °C. Subsequently, pods were removed and weighed separately, after which all seeds were removed from the pods and placed back in the drying oven for an additional 2 days. Total dry weight of the roots and the above ground tissue (without seeds) was used as an estimate of soy plant biomass. Dry mass of seeds was recorded, as a measure of seed yield.

After data recording, 9 random samples from each treatment were used to determine above ground total plant P concentrations. Oven dried samples were pooled in triplicate and ground using a coffee grinder to prepare samples for analysis. Total plant tissue P concentrations were determined using ICP-OES (inductively coupled plasma-optical emission spectroscopy), which was conducted by the Analytical Services Unit (ASU) at Queen’s University. Spinach (*Spinacia oleracea*) leaf tissue was used as an analytical reference, along with a separate P control sample. Above ground plant tissues analyzed included the stem, remaining leaves, and empty pods. Analysis of soluble Pi in soil samples collected from three random pots for treatments A-E was also done using ICP-OES. Prior to analysis, 0.5 g of each dried soil sample (oven dried at 70 °C for 2 days) was added to a glass vial with 40 mL of water. Sample vials were then placed on an orbital shaker overnight at 300 rpm to extract any soluble Pi. After shaking, the sample mixture was syringe-filtered (0.45 μm). Filtered solutions were then analyzed to determine Pi concentration using ICP-OES with a standard Pi control (ASU). For the plant growth and production experiments, as described earlier, statistical analysis was performed using R Studio (R version 3.3.2) and GraphPad Prism (version 7.0). One-way ANOVA with TukeyHSD post-hoc tests were used to determine any statistically significant differences between groups for final height, trifoliates produced, overall biomass, below and above ground biomass, and seed yield all using a significance level of 0.05.

### Accession codes

Biological sequencing data is available from NCBI Sequence Read Archive under the BioProject accession number: PRJNA544311.

## Supplementary information

Supplementary Information.
