# Supplementary material for: A practical assessment of nano-phosphate on soybean (Glycine max) growth and microbiome establishment
Source: Sci Rep. 2020 Jun 4;10:9151. doi: 10.1038/s41598-020-66005-w (PMC7272607; doi:10.1038/s41598-020-66005-w)
Supplement: Supplementary file 1 — Supplementary Information. [file 41598_2020_66005_MOESM1_ESM.docx]

**Supplementary Information**

**A practical assessment of nano-phosphate on soybean (*Glycine max*) growth and microbiome establishment**

Michelle M. McKnight^1^, Zhi Qu^1^, Julia K. Copeland^3^, David S. Guttman^3,4^, Virginia K. Walker^1,2^

^1^Department of Biology, Queen’s University, Kingston, ON

^2^Department of Biomedical and Molecular Sciences, Queen’s University, Kingston, ON

^3^Centre for the Analysis of Genome Evolution and Function, University of Toronto, ON

^4^Department of Cell and Systems Biology, University of Toronto, ON

**Article submitted to:** Scientific Reports

**Corresponding Author:** Michelle M. McKnight, MSc, Department of Biology, Queen’s University, 116 Barrie St., Kingston, ON, Canada, K7L 3N6; current address: Department of Biology, University of Waterloo, Waterloo, ON Canada

**E-mail:** [1mml1@queensu.ca](mailto:1mml1@queensu.ca)

**Additional supplementary microbiome experiment with soluble Pi treatment**

When the initial microbiome experiment was conducted, it consisted of five treatments: control (A), -P control (B), HA bulk control (C), 50 ppm nHA (D), and 100 ppm nHA (E). There was insufficient space to include a soluble Pi control treatment, so an additional supplementary experiment was conducted on a smaller scale under the same conditions used for the microbiome experiment including a soluble Pi treatment. Instead of including all five original treatments used in the initial microbiome experiment, this time only the -P control and a nHA treatment (100 ppm) were included alongside the soluble Pi treatment.

Soy plants were grown in SunGro Sunshine® Mix #2 and watered with Hoagland’s solution containing KNO_3_, as previously described for the microbiome experiment in the main manuscript_._ The soluble Pi treatment group, 1 mM Pi (supplied as 0.05 M Ca(H_2_PO_4_)_2_) was compared to the -P control (no added Pi) and nano-phosphate (nHA at 100 ppm or mg/kg) treatment groups in four replicates. One-way ANOVA and subsequent TukeyHSD post-hoc tests calculated through GraphPad Prism (version 7.0, GraphPad Software, La Jolla, CA, USA) were used to test for significant differences in pod number, seed number, and above- and below-ground biomass between treatment groups. Tests were conducted using a significance level of *P* < 0.05.

Growth trials conducted at 10 weeks showed that plants receiving soluble Pi had a significantly increased yield (10 seeds) compared with -P control and nHA treatments (3 seeds; *P* < 0.001). Additionally, above and below-ground biomass for soluble Pi plants was significantly greater than that of the nHA and -P control treatments, where above ground biomass was approximately 5-fold greater and below ground biomass was approximately three-fold greater with the addition of soluble Pi (*P* < 0.01, Table S1). This experiment demonstrates the capacity of the soy variety to show a healthy phenotype under non-limited Pi conditions, and also underscores the ineffectiveness of nHA to provide Pi as shown in the manuscript.
**Table S1** Biomass and yield from supplemental microbiome experiment with additional soluble Pi treatment.

| Treatment | Pod Number | Seed Number | Above-ground Biomass | Below-ground Biomass |
| --- | --- | --- | --- | --- |
| -P Control | **2.00** ± 0.82 | **3.00** ± 2.16 | **0.68** ± 0.38 | **0.64** ± 0.18 |
| nHA (100 ppm) | **2.25** ± 0.96 | **3.00** ± 1.41 | **0.82** ± 0.24 | **0.55** ± 0.09 |
| Soluble Pi | ***8.00** ± 0.82 | ***13.75** ± 3.20 | ***3.84** ± 0.26 | ***1.50** ± 0.34 |

* Post-hoc test shows significant difference between soluble Pi treatment and both -Pi control and nHA treatments (*P* > 0.01)

Figure S1 Average relative abundances of bacterial phyla across microbiome experiment treatment groups (A-E) from the endosphere/phyllosphere samples before (A) and after (B) removal of cyanobacteria/chloroplast phylum sequences. The V4 16S rRNA sequencing of DNA extracted from soybean leaf tissue, was conducted using PNA clamps, which have been reported to decrease the probability of chloroplast DNA amplification.

Figure S2 Principle coordinates analysis (PCoA) of fungal beta diversity in bulk soil and rhizosphere samples from microbiome experiment using the Bray-Curtis dissimilarity metric. Samples were normalized using both CSS normalization and rarefaction to the minimum sample count prior to beta diversity calculations.

Figure S3 Foliar damage to soybean trifoliate leaves and bacterial infection present on leaves – potentially introduced via thrips carrier, which occurred during greenhouse growth and production experiments (GP1 and GP2 replicates). Small juvenile thrip can be seen on the center vein of the middle trifoliate leaf, marked by red circle.

**Table S2** Number of OTUs and OTU reads of endosphere/phyllosphere microbiome experiment samples before and after filtering out chloroplast reads.

|  | *With Plastid Sequences* | *Plastid Sequences Removed* |
| --- | --- | --- |
| Number of samples | 50 | 50 |
| Number of OTUs | 929 | 926 |
| Total reads | **3,515,900** | **1,662** |
| Average reads per sample | 70,318 | 33.2 |
| Standard deviation | 31,030 | 33.9 |
| Min reads per sample | 8,980 | 1 |
| Max reads per sample | 138,675 | 196 |

**Table S3** Summary of rhizosphere phyla whose relative abundances were significantly different between microbiome experimental treatments.

| Phylum | Non-parametric ANOVA | | Post-hoc Test | |
| --- | --- | --- | --- | --- |
|  | chi-squared | *P*-value | Groups Compared | *P*-value adj. |
| Other | 17.2 | 1.78E-03 | A-C | 3.12E-03 |
|  |  |  | A-E | 2.27E-02 |
|  |  |  | A-D | 1.28E-02 |
|  |  |  | A-B | 2.04E-02 |
| Acidobacteria | 19.0 | 7.83E-04 | A-C | 2.27E-02 |
|  |  |  | B-C | 1.40E-03 |
|  |  |  | B-D | 4.12E-02 |
| Bacteroidetes | 19.6 | 5.87E-04 | A-C | 1.70E-03 |
|  |  |  | A-E | 3.30E-03 |
|  |  |  | A-D | 1.35E-02 |
| Proteobacteria | 11.1 | 2.56E-02 | A-D | 1.94E-02 |

Note: A = Control Treatment, B = -P Control Treatment, C = 50 ppm HA Treatment, D = 50 ppm nHA Treatment, and E = 100 ppm nHA Treatment

**Table S4** Summary of bacterial soil phyla whose relative abundances were significantly different between microbiome experiment treatments.

| Phylum | Non-parametric ANOVA | | Post-hoc Test | |  |
| --- | --- | --- | --- | --- | --- |
|  | chi-squared | *P*-value | Groups Compared | *P*-value adj. |  |
| Other | 21.4 | 2.59E-04 | A-C | 3.72E-03 | |
|  |  |  | A-E | 5.27E-03 | |
|  |  |  | A-D | 3.94E-04 | |
|  |  |  | A-B | 1.25E-02 | |
| Acidobacteria | 25.7 | 3.70E-05 | A-C | 3.73E-03 | |
|  |  |  | A-E | 1.25E-03 | |
|  |  |  | A-D | 1.03E+03 | |
|  |  |  | B-C | 3.93E-02 | |
|  |  |  | B-E | 1.58E-02 | |
| Armatimonadetes | 15.6 | 3.53E-03 | A-D | 2.39E-02 | |
|  |  |  | B-D | 4.12E-02 | |
| Bacteroidetes | 24.3 | 6.87E-05 | A-C | 4.19E-03 | |
|  |  |  | A-E | 4.50E-04 | |
|  |  |  | A-D | 1.75E-04 | |
| Gemmatimonadetes | 13.5 | 8.89E-03 | A-C | 1.15E-02 | |
| Planctomycetes | 10.8 | 2.84E-02 | C-E | 1.85E-02 | |
| WPS-1 | 12.3 | 1.50E-02 | A-E | 1.42E-02 | |
|  |  |  | A-D | 4.33E-02 | |

Note: A = Control Treatment, B = -P Control Treatment, C = 50 ppm HA Treatment, D = 50 ppm nHA Treatment, and E = 100 ppm nHA Treatment

**Table S5** Comparison of average bacterial beta diversity distances within a treatment to the average distance between treatments for soil samples in the microbiome experiment. Each analysis row represents a comparison of the average distance within the treatment (indicated in column 1) to the average distance between the treatment in column 1 and the treatment in column 2 for each of the five treatment sections.

| Distance within treatment comparison | Treatment for distance between comparison | CSS Normalized | | | Rarefied | | |
| --- | --- | --- | --- | --- | --- | --- | --- |
|  |  | ***t stat*** | ***P-Value*** | ***P-Adj.*** | ***t stat*** | ***P-Value*** | ***P-Adj.*** |
| Control | -P Control | -3.10 | 2.33E-03 | 3.17E-01 | -2.39 | 1.80E-02 | 1 |
|  | HA 50 ppm | -6.60 | 7.62E-10 | **1.04E-07*** | -7.01 | 8.80E-11 | **1.20E-08*** |
|  | nHA 50 ppm | -6.83 | 2.27E-10 | **3.09E-08*** | -6.23 | 4.99E-09 | **6.79E-07*** |
|  | nHA 100 ppm | -7.32 | 1.64E-11 | **2.23E-09*** | -6.29 | 3.56E-09 | **4.84E-07*** |
| -P Control | Control | -4.43 | 1.83E-05 | **2.49E-03** | -6.56 | 8.95E-10 | **1.22E-07** |
|  | HA 50 ppm | -3.44 | 7.54E-04 | 1.03E-01 | -4.27 | 3.58E-05 | **4.87E-03*** |
|  | nHA 50 ppm | -3.10 | 2.30E-03 | 3.13E-01 | -2.49 | 1.40E-02 | 1 |
|  | nHA 100 ppm | -3.28 | 1.30E-03 | 1.77E-01 | -1.98 | 4.98E-02 | 1 |
| HA 50 ppm | Control | -12.57 | 6.90E-25 | **9.39E-23*** | -10.90 | 1.66E-20 | **2.26E-18*** |
|  | -P Control | -11.59 | 2.54E-22 | **3.45E-20** | -4.17 | 5.16E-05 | **7.01E-03*** |
|  | nHA 50 ppm | -1.38 | 1.71E-01 | 1 | 0.43 | 6.65E-01 | 1 |
|  | nHA 100 ppm | -5.78 | 4.50E-08 | **6.12E-06** | -2.35 | 2.00E-02 | 1 |
| nHA 50 ppm | Control | -10.79 | 3.11E-20 | **4.23E-18*** | -9.62 | 3.30E-17 | **4.48E-15*** |
|  | -P Control | -7.56 | 4.46E-12 | **6.06E-10** | -2.38 | 1.86E-02 | 1 |
|  | HA 50 ppm | 1.09 | 2.76E-01 | 1 | 0.45 | 6.55E-01 | 1 |
|  | nHA 100 ppm | -3.04 | 2.86E-03 | 3.89E-01 | -0.99 | 3.24E-01 | 1 |
| nHA 100 ppm | Control | -10.34 | 4.56E-19 | **6.20E-17*** | -9.18 | 4.35E-16 | **5.92E-14*** |
|  | -P Control | -6.57 | 8.70E-10 | **1.18E-07** | -0.94 | 3.47E-01 | 1 |
|  | HA 50 ppm | -2.55 | 1.18E-02 | 1 | -1.48 | 1.42E-01 | 1 |
|  | nHA 50 ppm | -2.05 | 4.23E-02 | 1 | -0.22 | 8.24E-01 | 1 |

Significant *P*-values are in bold, with and * indicating significant difference comparing within and between treatment when using either of the two treatments being compared as the within treatment distance.

**Table S6** Weighted bacterial rhizosphere comparison of average beta diversity distances within a treatment to the average distance between treatments in the microbiome experiment. Each analysis row represents a comparison of the average distance within the treatment (indicated in column 1) to the average distance between the treatment in column 1 and the treatment in column 2 for each of the five treatment sections.

| Distance within treatment comparison | Treatment for distance between comparison | CSS Normalized | | | Rarefied | | |
| --- | --- | --- | --- | --- | --- | --- | --- |
|  |  | ***t stat*** | ***P-Value*** | ***P-Adj.*** | ***t stat*** | ***P-Value*** | ***P-Adj.*** |
| Control | -P Control | -3.51 | 6.01E-04 | 8.18E-02 | -3.03 | 2.91E-03 | 3.96E-01 |
|  | HA 50 ppm | -7.11 | 5.16E-11 | **7.02E-09*** | -5.40 | 2.75E-07 | **3.75E-05*** |
|  | nHA 50 ppm | -6.64 | 6.06E-10 | **8.24E-08*** | -8.82 | 3.64E-15 | **4.95E-13*** |
|  | nHA 100 ppm | -7.65 | 2.74E-12 | **3.73E-10*** | -5.31 | 4.15E-07 | **5.64E-05*** |
| -P Control | Control | -1.52 | 1.31E-01 | 1 | -5.52 | 1.58E-07 | **2.15E-05** |
|  | HA 50 ppm | -0.71 | 4.81E-01 | 1 | 0.28 | 7.83E-01 | 1 |
|  | nHA 50 ppm | -2.17 | 3.15E-02 | 1 | -3.22 | 1.61E-03 | 2.19E-01 |
|  | nHA 100 ppm | -0.52 | 6.05E-01 | 1 | -0.85 | 3.97E-01 | 1 |
| HA 50 ppm | Control | -10.21 | 1.03E-18 | **1.40E-16*** | -10.38 | 3.59E-19 | **4.88E-17*** |
|  | -P Control | -6.01 | 1.44E-08 | **1.96E-06** | -3.01 | 3.13E-03 | 4.26E-01 |
|  | nHA 50 ppm | -1.68 | 9.43E-02 | 1 | -2.32 | 2.19E-02 | 1 |
|  | nHA 100 ppm | -0.44 | 6.57E-01 | 1 | -1.17 | 2.42E-01 | 1 |
| nHA 50 ppm | Control | -4.87 | 2.94E-06 | **4.00E-04*** | -8.25 | 9.62E-14 | **1.31E-11*** |
|  | -P Control | -2.00 | 4.72E-02 | 1 | -0.49 | 6.26E-01 | 1 |
|  | HA 50 ppm | 1.83 | 6.92E-02 | 1 | 2.55 | 1.18E-02 | 1 |
|  | nHA 100 ppm | 1.41 | 1.62E-01 | 1 | 0.83 | 4.10E-01 | 1 |
| nHA 100 ppm | Control | -12.30 | 3.64E-24 | **4.95E-22*** | -8.24 | 1.01E-13 | **1.38E-11*** |
|  | -P Control | -7.15 | 4.06E-11 | **5.52E-09** | -1.43 | 1.55E-01 | 1 |
|  | HA 50 ppm | -1.33 | 1.85E-01 | 1 | 0.99 | 3.26E-01 | 1 |
|  | nHA 50 ppm | -3.27 | 1.34E-03 | 1.82E-01 | -2.37 | 1.92E-02 | 1 |

Significant *P*-values are in bold, with and * indicating significant difference comparing within and between treatment when using either of the two treatments being compared as the within treatment distance.

**Table S7** Summary of fungal phyla in soil whose average relative abundances were significantly different between microbiome experiment treatments.

| Phylum | Non-parametric ANOVA | | Post-hoc Test | |
| --- | --- | --- | --- | --- |
|  | chi-squared | *P*-value | Groups Compared | *P*-value adj. |
| Other | 17.6 | 1.46E-03 | A-C | 2.64E-02 |
|  |  |  | A-E | 3.30E-03 |
|  |  |  | A-D | 3.74E-02 |
| Ascomycota | 17.9 | 1.30E-04 | A-C | 2.05E-02 |
|  |  |  | A-D | 2.92E-02 |
|  |  |  | A-E | 2.61E-03 |
| Zygomycota* | 13.8 | 7.89E-04 | A-B | 3.93E-02 |
|  |  |  | A-C | 2.16E-02 |
|  |  |  | A-D | 4.12E-02 |
|  |  |  | A-E | 3.56E-02 |

Note: A = Control Treatment, B = -P Control Treatment, C = 50 ppm HA Treatment, D = 50 ppm nHA Treatment, and E = 100 ppm nHA Treatment

* Phylum present in community at <1% relative abundance

**Table S8** Summary of average relative abundances of fungal phyla in the rhizosphere that are significantly different between microbiome experiment treatments.

| Phylum | Non-parametric ANOVA | | Post-hoc Test | |
| --- | --- | --- | --- | --- |
|  | chi-squared | *P*-value | Groups Compared | *P*-value adj. |
| Other | 16.7 | 2.14E-03 | A-E | 5.90E-03 |
|  |  |  | B-E | 3.56E-02 |
| Ascomycota | 19.0 | 7.83E-04 | A-E | 6.61E-03 |
|  |  |  | B-E | 4.12E-02 |
| Zygomycota* | 17.7 | 1.40E-03 | B-E | 4.40E-04 |

Note: A = Control Treatment, B = -P Control Treatment, C = 50 ppm HA Treatment, D = 50 ppm nHA Treatment, and E = 100 ppm nHA Treatment

* Phylum present in community at <1% relative abundance

**Table S9** Fungal genera in soil with significant differences in average relative abundances between microbiome experiment treatments.

| Genus | Non-parametric ANOVA | | Post-hoc Test | |
| --- | --- | --- | --- | --- |
|  | chi-squared | *P*-value | Groups Compared | *P*-value adj. |
| Other | 17.6 | 1.46E-03 | A-C | 2.64E-02 |
|  |  |  | A-D | 3.74E-02 |
|  |  |  | A-E | 3.30E-03 |
| Ascomycota; Other* | 9.9 | 4.09E-02 | C-E | 4.30E-02 |
| Davidella | 13.4 | 9.48E-03 | B-C | 2.05E-02 |
|  |  |  | B-E | 3.56E-02 |
| Pseudogymnoascus* | 11.5 | 2.50E-02 | A-D | 2.80E-02 |
| Trichocomaceae; Other* | 12.6 | 1.36E-02 | A-D | 9.22E-03 |
| Aspergillus* | 13.4 | 9.58E-03 | A-D | 2.05E-02 |
| Eupenicillium | 11.7 | 1.97E-02 | A-D | 1.20E-02 |
| Paecilmyces | 17.7 | 1.43E-03 | A-C | 3.56E-02 |
|  |  |  | B-C | 6.98E-03 |
|  |  |  | B-D | 3.23E-02 |
| Pezizales* | 13.9 | 7.67E-03 | B-E | 2.86E-03 |
| Sphaerosphorella* | 15.6 | 3.54E-03 | A-E | 3.91E-02 |
|  |  |  | B-E | 8.68E-03 |
| Sarcoscypha* | 14.0 | 7.26E-03 | B-E | 1.67E-02 |
| Hypocrea | 22.3 | 1.73E-04 | A-D | 2.16E-02 |
|  |  |  | A-E | 8.07E-04 |
|  |  |  | B-E | 1.21E-02 |
| Gibberella | 10.5 | 3.34E-02 | B-C | 4.33E-02 |
| Humicola* | 10.5 | 3.25E-02 | C-E | 2.57E-02 |
| Neurospora* | 18.6 | 9.47E-04 | A-C | 8.70E-03 |
|  |  |  | B-C | 6.60E-03 |
|  |  |  | C-E | 4.10E-02 |
| Thamnidium* | 15.5 | 3.70E-03 | A-B | 1.58E-02 |
|  |  |  | A-C | 1.20E-02 |
|  |  |  | A-D | 3.40E-02 |
|  |  |  | A-E | 1.94E-02 |
| Zygorhynchus* | 14.1 | 6.90E-03 | A-C | 9.70E-03 |
|  |  |  | A-D | 4.54E-02 |
|  |  |  | A-E | 3.23E-02 |

Note: A = Control Treatment, B = -P Control Treatment, C = 50 ppm HA Treatment, D = 50 ppm nHA Treatment, and E = 100 ppm nHA Treatment

* Genus present in community at <1% relative abundance

**Table S10** Fungal genera in rhizosphere with significant differences in average relative abundances between microbiome experiment treatments.

| Genus | Non-parametric ANOVA | | Post-hoc Test | |
| --- | --- | --- | --- | --- |
|  | chi-squared | *P*-value | Groups Compared | *P*-value adj. |
| Other | 16.8 | 2.14E-03 | A-E | 5.90E-03 |
|  |  |  | B-E | 3.56E-02 |
| Ascomycota; Other* | 10.2 | 3.56E-02 | A-E | 3.92E-02 |
| Alternaria* | 10.6 | 3.19E-02 | B-E | 3.22E-02 |
| Aspergillus* | 18.6 | 9.52E-04 | A-C | 3.56E-02 |
|  |  |  | A-D | 1.21E-02 |
|  |  |  | B-D | 4.77E-02 |
| Baecilomyces* | 18.5 | 1.01E-03 | A-C | 5.60E-03 |
|  |  |  | A-D | 3.50E-03 |
|  |  |  | A-E | 5.30E-03 |
| Coniochaeta* | 14.7 | 5.35E-03 | A-D | 7.58E-03 |
| Lecythophora* | 10.7 | 3.04E-02 | B-E | 1.14E-02 |
| Hypocrea | 18.0 | 1.23E-03 | A-D | 3.50E-03 |
|  |  |  | A-E | 5.30E-03 |
|  |  |  | B-E | 2.05E-02 |
| Gibberella | 11.8 | 1.93E-02 | A-C | 2.51E-02 |
| Xylaria | 12.6 | 1.37E-02 | A-E | 3.23E-02 |
| Rhodotorula* | 10.7 | 2.94E-02 | C-E | 3.95E-02 |
| Spizellomyces* | 11.3 | 2.40E-02 | C-E | 4.02E-02 |
| Thamnidium* | 16.7 | 1.90E-03 | B-E | 4.20E-04 |
| Zygorhynchus* | 15.4 | 4.02E-03 | B-E | 2.94E-03 |

Note: A = Control Treatment, B = -P Control Treatment, C = 50 ppm HA Treatment, D = 50 ppm nHA Treatment, and E = 100 ppm nHA Treatment

* Genus present in community at <1% relative abundance

**Table S11** Results of non-parametric statistical tests adonis and ANOSIM on beta diversity distance matrices for both bulk soil and rhizosphere fungal communities in microbiome experiment using rarefied and CSS normalized OTU data.

| Normalization | Type | Test | Test statistic | P value |
| --- | --- | --- | --- | --- |
| Rarefaction | Soil | ANOSIM | 0.18 | 1.00E-03 |
|  |  | adonis | 0.24 | 1.00E-03 |
|  | Root | ANOSIM | 0.14 | 2.00E-03 |
|  |  | adonis | 0.29 | 3.00E-03 |
| CSS Normalization | Soil | ANOSIM | 0.23 | 1.00E-03 |
|  |  | adonis | 0.18 | 1.00E-03 |
|  | Root | ANOSIM | 0.25 | 1.00E-03 |
|  |  | adonis | 0.17 | 1.00E-03 |

**Supplementary Methods**

**Hoagland’s solution**

Hoagland’s nutrient solution was made following recipes from Hoagland and Arnon^1^ for solution without nitrogen, but also excluded the addition of Ca(H_2_PO_4_)_2_ as the nutrient solution was required to be both N and P deficient. To make 1 L of solution: 5 mL of 0.5 *M* K_2_SO_4_, 2 mL of 1.0 *M* MgSO_4_, 200 mL of 0.01 *M* CaSO_4_, 1 mL of 0.5% iron tartrate, and 1 mL of micronutrient solution (Table S12) were combined. 500 mL of ddH_2_O was added and pH of solution was adjusted to 6.0 using 0.1 *M* solution of H_2_SO_4_. After pH adjustment, ddH_2_O was added to reach final volume of 1 L.

**Table S12** Micronutrient solution components

| Compound | Formula | Concentration in Micronutrient Solution (g/L) |
| --- | --- | --- |
| Boric Acid | H_3_BO_3_ | 2.86 |
| Manganese Chloride | MnCl_2_ • H_2_O | 1.81 |
| Zinc Sulfate | ZnSO_4_ • 7H_2_O | 0.22 |
| Copper Sulfate | CuSO_4_ • 5H_2_O | 0.08 |
| Molybdic Acid | H_2_MoO_4_ • H_2_O | 0.02 |

**A**

**B**

Figure S4 Set-up of soy microbiome experiment (A) and for growth and production experiments (B). Three seeds and nHA/HA both placed 1 cm below the surface in 13 cm pot containing 86 g of the 5% farm soil mixture (A). nHA or HA was placed 7.6 cm below the surface of soil and 5.1 cm below soybean seeds as recommended by the OMAFRA for placement of solid fertilizer for growth experiment, with soybean seeds placed approximately 2.5 cm away from the centre of the pot (20 cm diameter), equidistant from each other (B).

**REFERENCES**

1.	Hoagland, D. R. & Arnon, D. I. The water-culture method for growing plants without soil. *Circ. Calif. Agric. Exp. Stn.* **347**, (1950).
